# Treatment of acute hand ischemia following a scald injury in a hemodialysis patient with central venous occlusion: a case report

**DOI:** 10.3389/fmed.2026.1845565

**Published:** 2026-07-02

**Authors:** Wei Huang, Qi-qi Wang, Yu Xie, Ren-song Yue

**Affiliations:** 1 Department of Vascular Surgery, Hospital of Chengdu University of Traditional Chinese Medicine, Chengdu, Sichuan, China; 2 Clinical Medical School, Chengdu University of Traditional Chinese Medicine, Chengdu, China; 3 Hospital of Chengdu University of Traditional Chinese Medicine, Chengdu, Sichuan, China

**Keywords:** access-induced hand ischemia (AIHI), arteriovenous fistula, case report, central venous occlusion, dialysis access

## Abstract

**Objective:**

This case report describes a rare presentation of acute digital ischemia in a hemodialysis patient, precipitated by central venous occlusion (CVO). We reveal a unique pathological mechanism: venous hypertension induced by CVO and subsequent forearm edema lead to distal venous congestion and secondary hypoperfusion of the distal limb.

**Case report:**

A 56-year-old female on long-term maintenance hemodialysis presented with progressive left forearm swelling and acute digital ischemia following a minor wrist scald. Clinical examination revealed severe pain, numbness, and cyanosis of the digits. Duplex ultrasonography demonstrated massive subcutaneous edema of the forearm and diminished flow in the ulnar artery, alongside a chronically occluded distal radial artery at the arteriovenous fistula (AVF) site. Previous imaging had confirmed ipsilateral subclavian vein occlusion. Given the threat to limb viability, emergency AVF ligation was performed under local anesthesia to reduce forearm venous pressure and increase ulnar artery blood flow. Immediate postoperative recovery was observed, with rapid restoration of digital skin temperature, capillary refill, and resolution of forearm edema.

**Conclusion:**

In hemodialysis patients with confirmed central venous obstruction (CVO), significant limb swelling can trigger a reduction in distal limb perfusion, leading to severe ischemic symptoms. Early recognition of this venous-hypertensive mechanism is critical; timely intervention, such as AVF ligation, can reverse ischemia and prevent permanent tissue loss.

## Introduction

Access-induced hand ischemia (AIHI) is a severe complication affecting approximately 5% of patients with arteriovenous fistulas (AVFs) or grafts (AVGs) (1). The primary pathophysiology involves compromised distal arterial perfusion caused by a pressure gradient across the anastomosis. This is often exacerbated by proximal inflow disease, distal outflow obstruction, excessive fistula flow, or catheter-related competitive flow (2). While central venous occlusion (CVO) is a well-recognized cause of venous hypertension and limb swelling, reports of AIHI triggered specifically by CVO remain scarce. To our knowledge, this is the first reported case of acute digital ischemia secondary to scald injury-induced venous hypertension in the setting of chronic CVO.

## Case report

**Figure 1:**
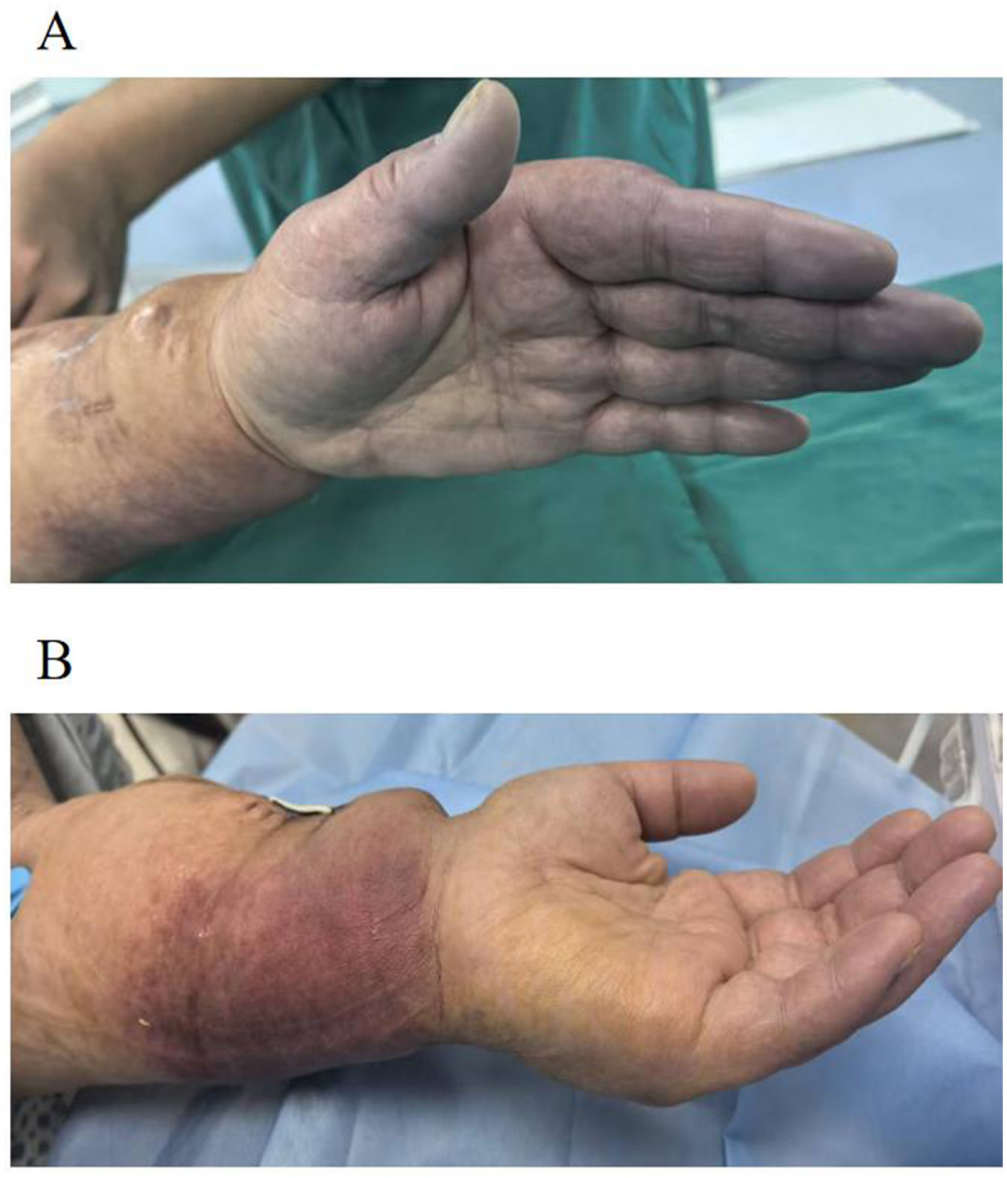
**(A)** Left-sided cyanosis of the finger, **(B)** Erythema and swelling of the ulnar aspect of the left wrist.

A 56-year-old female with end-stage renal disease (ESRD) on maintenance hemodialysis presented with acute limb-threatening ischemia. Two days after sustaining a scald injury to the ulnar aspect of the left wrist, the patient experienced rapidly progressing erythema and swelling of the left forearm, accompanied by severe pain, cyanosis, and numbness of the fingers (Figure 1A). Physical examination revealed an area of erythema and swelling measuring approximately 5 × 8 cm on the ulnar side of the left forearm, with no blister formation. The fingers were cold and cyanotic, with absent capillary refill. Notably, the symptoms of pain and cyanosis neither improved nor worsened regardless of whether the limb was elevated or placed in a dependent position (Figure 1B).

Bedside Doppler ultrasonography revealed severe edema of the ulnar subcutaneous tissue of the forear, occlusion of the distal radial artery segment of the AVF, proximal ulnar artery peak systolic velocity: 25 cm/s; distal peak systolic velocity: 20 cm/s, Continuous flow was visualized within the ulnar artery, with no intraluminal filling defects. Serum myoglobin was elevated at 220 μg/L, suggestive of early muscle injury.

**Figure 2:**
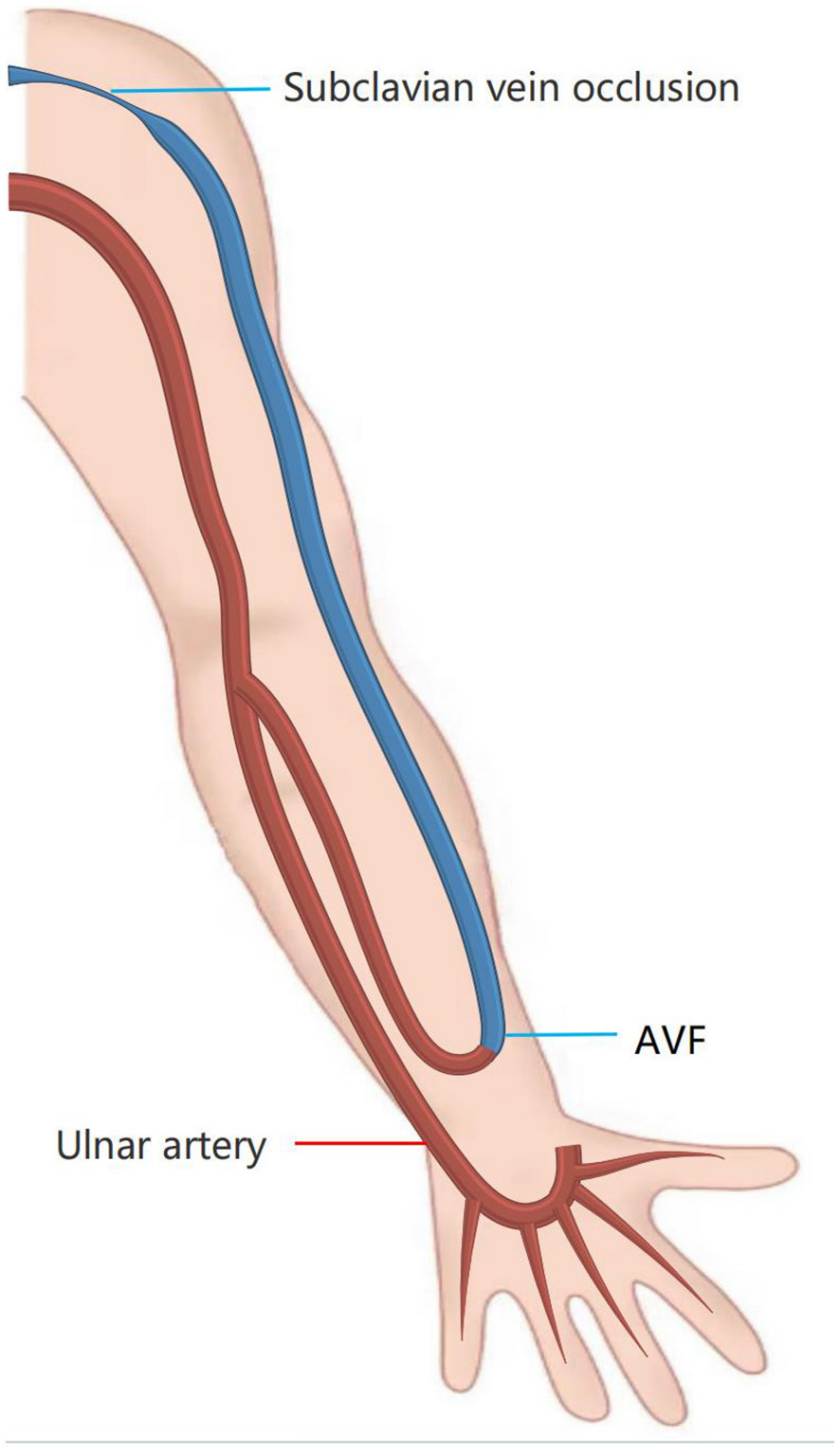
Schematic representation of blood flow configuration in the left upper extremity autologous AVF.

**Figure 3:**
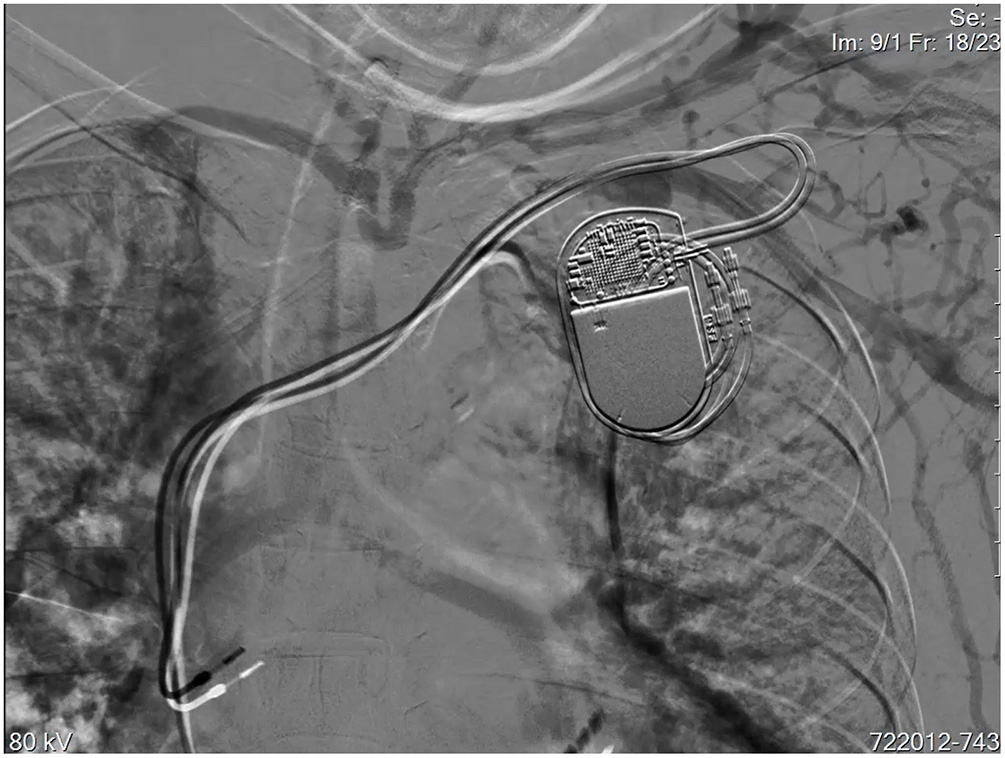
Left subclavian vein occlusion.

Her surgical history was significant for a left forearm arteriovenous fistula (AVF) created 8 years prior, which required reconstruction via a radial artery-to-cephalic vein end-to-end anastomosis 6 years prior following an anastomotic occlusion; consequently, the left hand relied primarily on the ulnar artery for its blood supply (Figure 2). Additionally, she had a cardiac pacemaker implanted 5 years prior. Six months before the current presentation, the patient developed left upper limb edema. Venography at that time confirmed CVO involving the subclavian veins (Figure 3). Although symptoms transiently improved following balloon angioplasty, the edema recurred 2 months later and persisted without further intervention.

**Figure 4:**
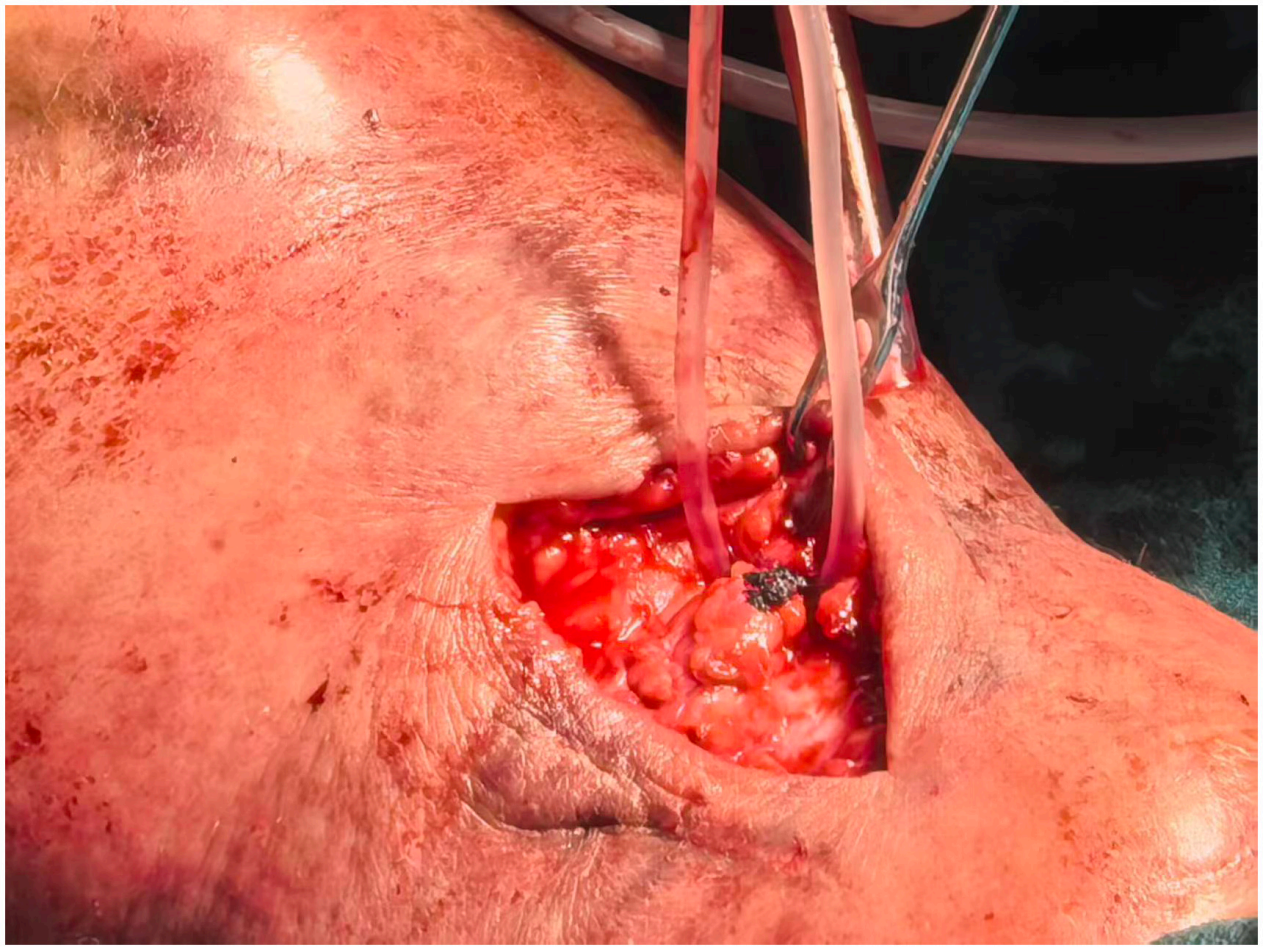
Under local anesthesia, ligate the junction of the cephalic vein at the anastomosis of the arteriovenous fistula.

**Figure 5:**
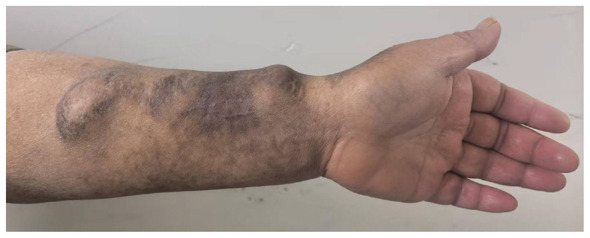
Postoperative left finger skin color and temperature returned to normal.

The combined clinical sequence of chronic subclavian vein occlusion, a subsequent scald injury, and resultant severe forearm edema led to a definitive diagnosis of acute digital ischemia. Emergency ligation of the AVF was performed under local anesthesia. Intraoperative findings showed marked improvement in forearm swelling following ligation of the arteriovenous fistula (Figure 4). Postoperatively, there was immediate and dramatic improvement: finger perfusion was restored, cyanosis resolved, skin temperature normalized, and the severe forearm swelling subsided (Figure 1B). The patient reported complete relief of pain and numbness. Follow-up ultrasonography 24 h post-operatively showed a peak systolic velocity (PSV) of 47 cm/s in the ulnar artery. Following the emergency procedure, a tunneled cuffed catheter was inserted into the right internal jugular vein for interim hemodialysis. Once stabilized, the patient subsequently underwent the creation of a new arteriovenous fistula in the right upper limb. At the 1-month postoperative follow-up, the patient exhibited increased pigmentation of the left forearm, though swelling had significantly improved. The skin temperature and color of the left hand were normal, and the sensory and motor functions of the fingers remained intact (Figure 5).

## Discussion

This case illustrates a critical, albeit rare, complication of hemodialysis access in the setting of CVO. The underlying subclavian vein occlusions established a baseline of chronic venous hypertension. Venous hypertension combined with forearm edema can significantly increase forearm venous congestion and secondary limb hypoperfusion (1).

Owing to central venous occlusion, the patient had a history of chronic forearm swelling. With the distal radial artery occluded, the limb relied primarily on the ulnar artery for blood supply, yet no symptoms of steal syndrome had previously manifested. In this case, Local first-degree burns serve as a precipitating factor that exacerbates forearm edema. When severe edema leads to a massive accumulation of fluid within the interstitial space, the interstitial fluid pressure (IFP) rises significantly. Tissue perfusion, on the other hand, depends on the balance between systemic blood pressure and tissue microvascular resistance. The walls of capillaries and venules in the microcirculation are extremely thin, and their intraluminal pressure is low (with the distal capillary pressure being approximately 15–20 mmHg, and venule pressure being even lower). When the interstitial pressure exceeds the intraluminal pressure of these microvessels, the vessel lumens undergo mechanical compression, becoming narrowed or even occluded. This luminal compression causes a sharp increase in the local microvascular resistance, which subsequently leads to a compensatory rise in intracapillary pressure. This not only accelerates fluid exudation into the interstitial space (further aggravating the edema) but also causes extreme deceleration of local blood flow. Consequently, the effective perfusion pressure gradient between arterioles and venules narrows significantly, reducing the number of open capillary beds and causing a precipitous drop in blood flow, which ultimately triggers tissue ischemia (3).

The reported incidence of severe access-related ischemia requiring intervention is approximately 1.9%. Patients with upper-arm AVGs carry the highest risk (2.2%−2.8%), and females exhibit a 3.64-fold increased susceptibility (4). This patient's history of pacemaker implantation is a well-documented risk factor for subclavian vein stenosis or occlusion, which served as the pathophysiological foundation for this event (5, 6).

The elevated serum myoglobin level (220 μg/L) upon admission provided definitive biochemical evidence of muscle ischemia and served as a high-alert indicator for the potential development of rhabdomyolysis. Ultrasound demonstrates that the ulnar artery lumen is patent with slow blood flow and no evidence of thromboembolism. The patient has a cardiac pacemaker lead in the occluded segment of the subclavian vein, which is a contraindication for stent implantation; furthermore, upper extremity edema persisted even after a previous percutaneous transluminal balloon angioplasty. Surgical recanalization of the subclavian vein is unlikely to provide rapid relief for the patient's upper extremity edema and ischemic symptoms. Given the severity of the compromise, emergency AVF ligation proved life-saving. By immediately ceasing the high-flow arterial input into the congested and obstructed venous system, the surgical procedure effectively relieved the patient's left forearm edema, reduced interstitial fluid pressure and microvascular resistance, increased ulnar artery blood flow, and successfully restored digital perfusion.

Arteriovenous (AV) access-induced peripheral ischemia includes: Type I: Retrograde Flow (True Steal); Type II: Inflow Stenosis; and Type III: Poor Collateral Circulation. Management of Type I requires addressing the access shunt; Type II requires prioritizing the relief of inflow stenosis; and Type III necessitates an assessment of collateral circulation reserve (2). The patient in this case presents with distal radial artery occlusion but has no prior history of digital ischemia. The primary cause of the current digital ischemia is severe forearm edema, which rapidly elevated interstitial fluid pressure and microvascular resistance, leading to a critical reduction in ulnar artery blood flow and digital perfusion. Reducing fistula flow alone could not rapidly alleviate forearm swelling or relieve ulnar artery compression. Arterial ultrasonography ruled out ulnar artery thrombosis and emergency ligation of the arteriovenous fistula proved to be a definitive and immediately effective intervention, as it eliminated forearm venous hypertension, enabling rapid decompression, restoration of ulnar artery blood flow, and reperfusion of the hand (7).

The rapid resolution of symptoms and signs post-ligation unequivocally confirmed the efficacy of this approach. Severe forearm edema complicated by ischemia may lead to acute compartment syndrome, potentially resulting in irreversible neuromuscular injury, Volkmann contracture, permanent functional impairment, as well as systemic complications such as rhabdomyolysis and acute kidney injury. The observed digital cyanosis and elevated myoglobin level at presentation indicated that without urgent decompression (achieved here via AVF ligation), the patient faced a high risk of digit or limb loss and potentially life-threatening systemic sequelae.

## Study limitations

Given the severity of the patient's clinical presentation and the urgency of the condition, upper extremity arteriography was not performed to evaluate the upper limb arteries and distal vasculature, or to further elucidate the cause of acute digital ischemia radiologically.

## Conclusion

Increased venous pressure resulting from central venous occlusion, combined with a localized thermal injury, may compromise distal limb perfusion. This condition presents similarly to, yet is distinct from, access-related steal syndrome. Emergency AVF ligation serves as an effective intervention, providing immediate reduction of venous hypertension, increasing ulnar artery blood flow, and prevention of permanent functional loss and amputation.

## Data Availability

The original contributions presented in the study are included in the article/supplementary material, further inquiries can be directed to the corresponding author.
